# Increased Behavioral and Neuronal Responses to a Hallucinogenic Drug in PACAP Heterozygous Mutant Mice

**DOI:** 10.1371/journal.pone.0089153

**Published:** 2014-02-20

**Authors:** Keisuke Hazama, Atsuko Hayata-Takano, Kazuki Uetsuki, Atsushi Kasai, Naoki Encho, Norihito Shintani, Kazuki Nagayasu, Ryota Hashimoto, Dora Reglodi, Tsuyoshi Miyakawa, Takanobu Nakazawa, Akemichi Baba, Hitoshi Hashimoto

**Affiliations:** 1 Laboratory of Molecular Neuropharmacology, Graduate School of Pharmaceutical Sciences, Osaka University, Suita, Osaka, Japan; 2 Molecular Research Center for Children’s Mental Development, United Graduate School of Child Development, Osaka University, Kanazawa University, Hamamatsu University School of Medicine, Chiba University and University of Fukui, Suita, Osaka, Japan; 3 Interdisciplinary Program for Biomedical Sciences, Institute for Academic Initiatives, Osaka University, Suita, Osaka, Japan; 4 iPS Cell-based Research Project on Brain Neuropharmacology and Toxicology, Graduate School of Pharmaceutical Sciences, Osaka University, Suita, Osaka, Japan; 5 Department of Psychiatry, Osaka University Graduate School of Medicine, Suita, Osaka, Japan; 6 Department of Anatomy, PTE-MTA “Lendület” PACAP Research Team, University of Pécs, Faculty of Medicine, Pécs, Hungary; 7 Division of Systems Medical Science, Institute for Comprehensive Medical Science, Fujita Health University, Toyoake, Aichi, Japan; 8 School of Pharmacy, Hyogo University of Health Sciences, Kobe, Hyogo, Japan; University of Rouen, France, France

## Abstract

Accumulating evidence from human genetic studies implicates the pituitary adenylate cyclase-activating polypeptide (PACAP) gene as a risk factor for psychiatric disorders, including schizophrenia and stress-related diseases. Mice with homozygous disruption of the PACAP gene display profound behavioral and neurological abnormalities that are ameliorated with the atypical antipsychotic and dopamine D_2_ and serotonin (5-HT)_2_ antagonist risperidone and the 5-HT_2_ receptor antagonist ritanserin; however, the underlying mechanisms remain unknown. Here, we investigated if PACAP heterozygous mutant (PACAP^+/−^) mice, which appear behaviorally normal, are vulnerable to aversive stimuli. PACAP^+/−^ mice were administered a 5-HT_2_ receptor agonist, (±)-2,5-dimethoxy-4-iodoamphetamine (DOI), a hallucinogenic drug, and their responses were compared with the littermate wild-type mice. After DOI injection, PACAP^+/−^ mice showed increased head-twitch responses, while their behavior was normal after saline. DOI induced deficits in sensorimotor gating, as determined by prepulse inhibition, specifically in PACAP^+/−^ mice. However, other 5-HT_2_ receptor-dependent responses, such as corticosterone release and hypothermia, were similarly observed in PACAP^+/−^ and wild-type mice. c-Fos expression analysis, performed in various brain regions, revealed that the DOI-induced increase in the number of c-Fos-positive cells was more pronounced in 5-HT_2A_ receptor-negative cells in the somatosensory cortex in PACAP^+/−^ mice compared with wild-type mice. These results indicate that PACAP^+/−^ mice exhibit specific vulnerability to DOI-induced deficits in cortical sensory function, such as exaggerated head-twitch responses and sensorimotor gating deficits. Our findings provide insight into the neural mechanisms underlying impaired behavioral responses in which 5-HT_2_ receptors are implicated.

## Introduction

Pituitary adenylate cyclase-activating polypeptide (PACAP) is a neuropeptide with multiple roles, including neurotransmitter, neuromodulator and neurotrophic factor [1]. Our recent studies have suggested that PACAP is associated with psychiatric disorders, including schizophrenia. Genetic variants of the PACAP gene that are overrepresented in schizophrenia patients are associated with reduced hippocampal volume and impaired memory performance [2]. A copy number gain of the PACAP gene due to a partial trisomy has been shown to cause severe mental retardation [3]. PACAP-deficient mice exhibit remarkable behavioral changes related to psychosis, memory impairment and depression that can be treated with the atypical antipsychotic and mixed D_2_ and serotonin (5-HT)_2_ antagonist risperidone and the 5-HT_2_ receptor antagonist ritanserin [4]–[9]. In addition, Vacic et al. [10] found a significant association of copy number gains at chromosome 7q36.3 with schizophrenia, which results in increased expression of the common VIP and PACAP receptor VPAC2 in cultured lymphocytes. Furthermore, Ressler et al. [11], [12] demonstrated a sex-specific association of post-traumatic stress disorder (PTSD) with PACAP and the PACAP-selective receptor PAC_1_ in females. These studies provide convergent evidence for psychiatric implications of the PACAP signaling system; however, the underlying mechanisms remain unknown.

There is a great deal of evidence implicating 5-HT_2_ receptors in various neurological and psychiatric conditions. Hallucinogenic drug-induced activation of 5-HT_2_ receptors is closely related to their reinforcing and/or aversive effects [13]. Impaired 5-HT_2A_ receptor signaling plays a major role in schizophrenic episodes. Almost all currently available atypical antipsychotic drugs are 5-HT_2A_ receptor inverse agonists, as well as dopamine D_2_ receptor antagonists or partial agonists [14]. The attenuation of extrapyramidal symptoms by atypical antipsychotic drugs has been proposed to be mediated by dopamine release in the striatum induced by 5-HT_2A_ antagonism [15]. Furthermore, in a study using 5-HT_2A_ conditional knockout mice, a specific role for cortical 5-HT_2A_ receptor function in the modulation of conflict anxiety was observed, consistent with the hypothesized “top-down” control model of anxiety-related processes [16].

To provide insight into the neural mechanisms underlying impaired behavioral responses in which 5-HT_2_ receptors are implicated, we examined if PACAP mutant (PACAP^+/−^) mice, which appear behaviorally normal, are vulnerable to aversive stimuli; in this case, injection of the hallucinogenic 5-HT_2_ receptor agonist (±)-2,5-dimethoxy-4-iodoamphetamine (DOI). We also performed c-Fos expression analysis to identify brain regions with an altered response to DOI in PACAP^+/−^ mice.

## Materials and Methods

### Animals

All animal care and handling procedures were performed according to the Guidelines for the Care and Use of Laboratory Animals approved by the Japanese Pharmacological Society, and were approved by the Animal Care and Use Committee of the Graduate School of Pharmaceutical Sciences, Osaka University. All efforts were made to minimize the number of animals used.

Wild-type control (PACAP^+/+^) and PACAP^+/−^ mice were obtained by crossing female PACAP^+/+^ or PACAP^+/−^ mice on the C57BL/6J mouse background, and male PACAP^+/−^ mice on the 129S6/SvEvTac background. The generation of PACAP^+/−^ mice by gene targeting has been reported previously [5]. These mice were backcrossed at least 10 times with C57BL/6J or 6 times with 129S6/SvEvTac, which are statistically expected to be >99.90% and 98.44% congenic, respectively [6]. C57BL/6J mice were purchased from Shimizu Laboratory Supplies (Kyoto, Japan), and 129S6/SvEvTac mice were purchased from Taconic (Germantown, NY). All experiments were conducted with naïve 8–9-week-old male mice, group-housed (4–5 per cage) with a 12-h light–dark cycle (light on at 8∶00 am) at controlled room temperature (22±1°C). Pelleted food (CMF, Oriental Yeast, Osaka, Japan) and water were available *ad libitum*.

### Behavioral Analysis

Each behavioral study was performed using a separate cohort of mice. For assessment of the head-twitch response, mice were individually placed in observation cages (19×10×11 cm) for a 60-min habituation period. They were then intraperitoneally injected with either saline or DOI (Sigma–Aldrich, Tokyo, Japan), which were prepared just before use, and recordings were made for a duration of 60 min. Scoring began immediately after injection by trained blind observers. The head-twitch response is a distinctive paroxysmal head-twitching behavior that is easily distinguished from head-bobbing, lateral movements of the head and grooming.

Acoustic startle responses for the prepulse inhibition (PPI) experiment were measured in a startle chamber (SR-LAB; San Diego Instruments, San Diego, CA) using standard methods described previously [17]. Animals were placed in the startle chamber 5 min after intraperitoneal injection of DOI (1.0 mg/kg). The testing session started with 5 min of acclimatization to the startle chamber in the presence of 65 dB background broadband (white) noise. Testing consisted of forty 120 dB pulses alone and 10 pulses preceded (100 ms) by a prepulse of 68, 71 or 74 dB. Pulses were randomly presented with an average interval of 15 s between pulses. Twelve no-stimulus trials were included to assess spontaneous activity during testing. PPI was calculated as a percentage score: PPI (%) = (1 − (startle response for pulse with prepulse)/(startle response for pulse alone))×100.

### Immunohistochemistry and Quantitative Analysis

Immunohistochemistry for c-Fos was performed as described previously [18]. Briefly, mice were intraperitoneally injected with DOI and placed back into their home cages. Two hours after injection, mice were deeply anesthetized with 50 mg/kg pentobarbital, and perfused transcardially with saline followed by 4% paraformaldehyde in phosphate-buffered saline. Whole brains were dissected and postfixed in the same fixative overnight at 4°C. Then, brain blocks were cryoprotected in 20% sucrose in phosphate-buffered saline for 48 h at 4°C. For c-Fos staining, coronal brain sections (20-µm-thick) were prepared, and processed by immunohistochemistry using anti-c-Fos rabbit polyclonal primary antibody (1∶2 000 dilution; sc-52; Santa Cruz Biotechnology, Santa Cruz, CA) and biotin-labeled anti-rabbit IgG secondary antibody (1∶200 dilution; Nichirei, Tokyo, Japan).

The brain regions and the dimensions of the areas analyzed were as follows (Fig. S1): medial prefrontal cortex (mPFC; 500 µm×500 µm), core of the accumbens nucleus (Acb core; 250 µm×250 µm), shell of the accumbens nucleus (Acb shell; 250 µm×250 µm), somatosensory cortex (SSCx; 250 µm×250 µm, 3 positions), dorsolateral caudate putamen (DL-CPu; 500 µm×500 µm), dorsomedial caudate putamen (DM-CPu; 500 µm×500 µm), ventrolateral caudate putamen (VL-CPu; 500 µm×500 µm), ventral pallidum (VP; 500 µm×200 µm), basolateral nuclei of the amygdala (BLA; 300 µm×100 µm), lateral globus pallidus (LGP; 700 µm×300 µm), mediodorsal thalamic nucleus (MD; 200 µm×200 µm), paraventricular hypothalamic nucleus (PVN; 100 µm×100 µm, 3 positions), the CA1 field of the hippocampus (CA1; 300 µm×75 µm, 3 positions), granule cell layer of the dentate gyrus (GrDG; 150 µm×75 µm, 3 positions), polymorph layer of the dentate gyrus (PoDG; 300 µm×75 µm) and substantia nigra pars reticulata (SNR; 500 µm×200 µm). Both right and left hemispheres of three sections for each region selected were examined for counting c-Fos-positive cells in the areas of interest.

For double-immunofluorescence staining, sections were incubated with anti-c-Fos goat polyclonal antibody (1∶1 000 dilution; Santa Cruz) and anti-5-HT_2A_ receptor rabbit polyclonal antibody (1∶300 dilution; Abcam, Cambridge, MA), and then with Alexa Fluor 488-conjugated chicken anti-goat IgG (1∶1 000 dilution; Invitrogen, Eugene, OR) and Alexa Fluor 594-conjugated donkey anti-rabbit IgG (H+L) (1∶500 dilution; Invitrogen). Double-immunofluorescence-stained slices were photographed using a fluorescence microscope (Biozero BZ-9000; Keyence, Osaka, Japan), and positive cells were counted by experienced observers blinded to mouse genotype and treatment.

### Statistics

All data are expressed as the mean ± standard error of the mean (S.E.M.). Student’s *t*-test, one-way analysis of variance (ANOVA) followed by Dunnett’s test, or two-way ANOVA followed by the Tukey-Kramer test were used to assess statistical significance as appropriate. Data for open field test and head-twitch response were analyzed using two-way ANOVA for genotype as the intersubject factor and repeated measures with time as the intrasubject factor. Data for PPI were analyzed using three-way ANOVAs (genotype and treatment as the intersubject factors, and prepulse intensity as the intrasubject factors). Multiple comparisons were performed using the Student-Newman-Keuls test. A *p-*value lower than 0.05 was considered statistically significant. The statistical analyses were performed using a software package (StatView® 5.0 for Windows; SAS Institute, Cary, NC).

## Results

### Behavioral Abnormalities in PACAP^+/−^ Offspring from Maternal PACAP^+/−^ Mating, not from Maternal PACAP^+/+^ Mating

Because genetic background may account for behavioral variation [19], we first investigated locomotor activity in PACAP^+/−^ mice on the C57BL/6J–129S6/SvEvTac F_1_ mixed genetic background (B6–129S6) in the open field test. To obtain B6–129S6 F_1_ mice, male PACAP^+/−^ mice on the 129S6/SvEvTac background were mated with female PACAP^+/+^ or PACAP^+/−^ mice on the C57BL/6J background. F_1_ PACAP^+/−^ mice from PACAP^+/+^ dams did not exhibit hyperactivity or differences in rearing time or time spent in the center of the field in the open field (Fig. S2A, C, E). Repeated two-way ANOVA revealed no significant main effect of genotype [F_(1, 295)_ = 0.005, *p* = 0.95]. In contrast, F_1_ PACAP^+/−^ mice from PACAP^+/−^ dams showed significant increases in locomotor activity compared with PACAP^+/+^ littermates (Fig. S2B). Repeated two-way ANOVA revealed a significant main effect of genotype [F_(1, 170)_ = 5.93, *p*<0.05]. Concomitant with the increase in locomotor activity, F_1_ PACAP^+/−^ mice from PACAP^+/−^ dams showed significant increases in rearing time and time spent in the center of the field compared with PACAP^+/+^ littermates (Fig. S2D, F).

### Altered Behavioral Response to the 5-HT_2A_ Agonist DOI in PACAP^+/−^ Mice

**Figure 1:**
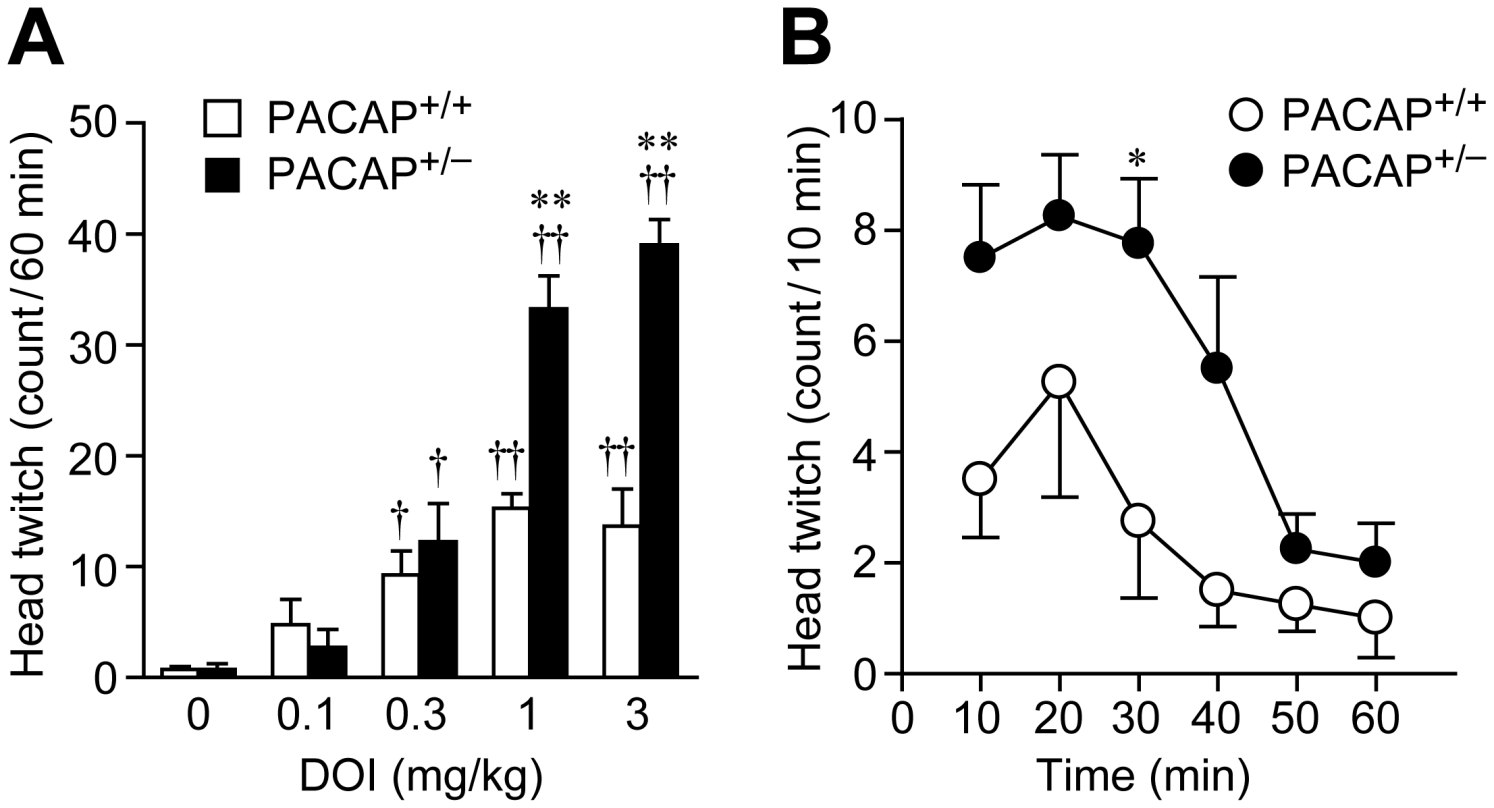
Effect of DOI on the head-twitch response in PACAP^+/−^ mice. (A) PACAP^+/+^ (open columns) and PACAP^+/−^ (closed columns) mice were treated with the indicated doses of DOI or saline. Head-twitch responses were counted over a 60-min period. (B) Time course of the effect of 1 mg/kg DOI administration on PACAP^+/+^ (open circles) and PACAP^+/−^ (closed circles) mice. Values are expressed as the mean ± SEM (n = 3–4). Statistically significant differences were assessed by two-way ANOVA followed by Tukey-Kramer test. *p<0.05, **p<0.01 vs. PACAP ^+/+^ mice; ^†^ *p*<0.05, ^††^ *p*<0.01 vs. saline.

To investigate the mechanisms by which susceptibility genes alter neural responses, we used PACAP^+/−^ mice obtained from PACAP^+/+^ dams, which display behaviors undistinguishable from their PACAP^+/+^ littermates, for the experiments that follow. To examine whether the behavioral response to external stimuli is altered in PACAP^+/−^ mice, we examined the DOI-induced head-twitch response, which is a characteristic head-shaking movement induced by the hallucinogenic drug via stimulation of 5-HT_2_ receptors [20]. The total number of head-twitch responses induced by DOI during a 60-min period was significantly increased in both genotypes in a dose-dependent manner, but the response in PACAP^+/−^ mice occurred significantly more frequently than in their PACAP^+/+^ littermates with doses of DOI (1.0 or 3.0 mg/kg) (Fig. 1A). Two-way ANOVA revealed a significant main effect of genotype [F_(1, 28)_ = 39.6, *p*<0.001] and DOI dose [F_(4, 28)_ = 55.8, *p*<0.001], and there was a significant interaction between genotype and treatment [F_(4, 28)_ = 14.1, *p*<0.001]. The maximal head-twitch response induced by 1.0 mg/kg DOI was elicited within approximately 20 minutes in both PACAP^+/+^ and PACAP^+/−^ mice (Fig. 1B).

**Figure 2:**
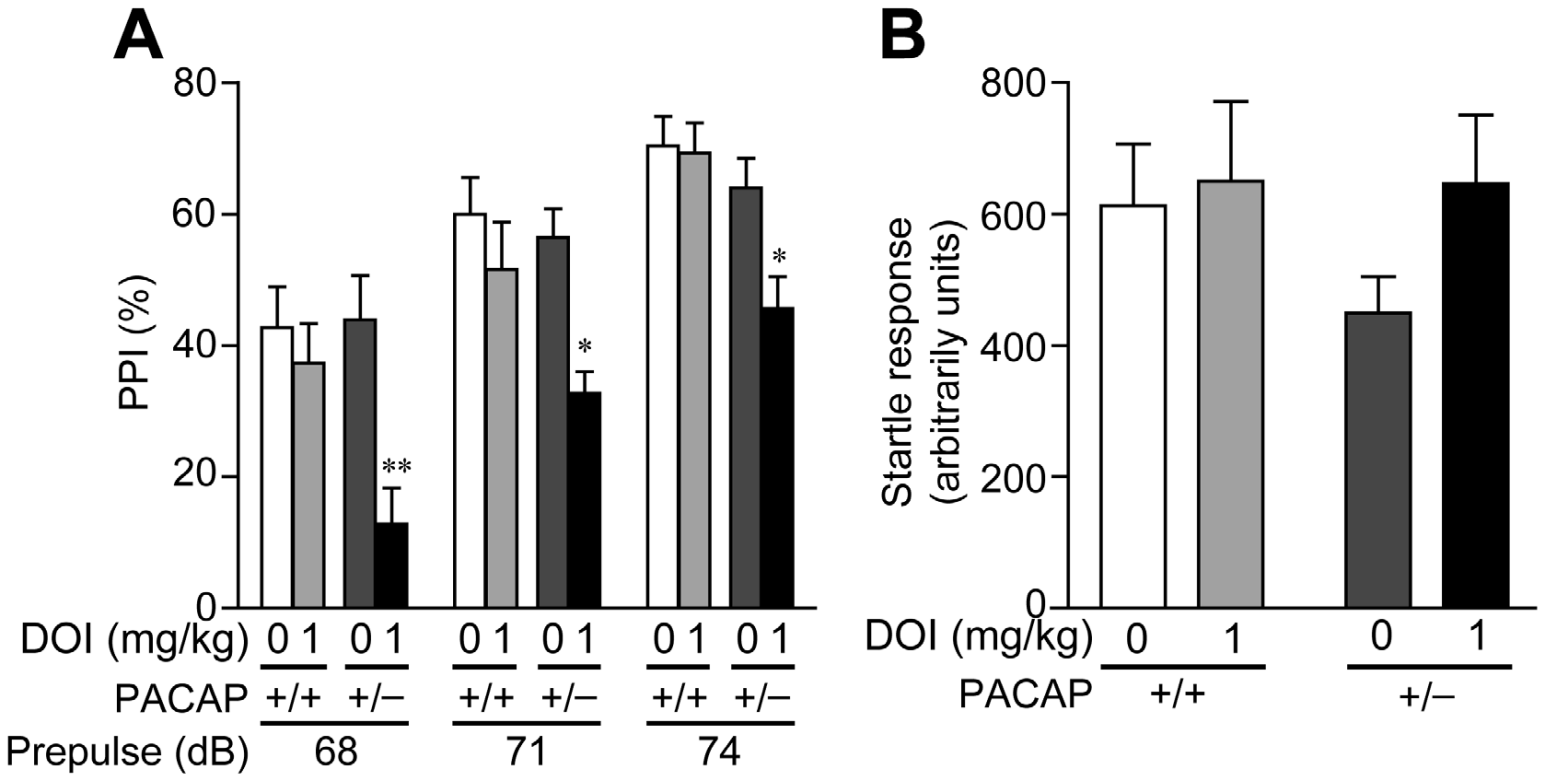
DOI-induced PPI deficits in PACAP^+/−^ mice. Effects of DOI on PPI (A) and acoustic startle response (B) were examined in PACAP^+/+^ (+/+) and PACAP^+/−^ (+/−) mice. DOI (1 mg/kg) was injected intraperitoneally 5 min before the experiments. Values are expressed as the mean ± SEM (n = 6–9). Differences were assessed with repeated three-way ANOVA with post hoc Tukey-Kramer test. *p<0.05, **p<0.01 vs. saline.

PPI is a reliable, robust quantitative phenotype that is useful for probing the neurobiology and genetics of gating deficits in schizophrenia across species [21]. DOI is known to disrupt PPI via 5-HT_2A_ receptors in rodents [22]. We therefore examined PPI in PACAP^+/+^ and PACAP^+/−^ mice treated with or without DOI. There was no difference in PPI levels between the two genotypes of mice following saline injection (Fig. 2A). Although 1.0 mg/kg DOI had no effect in wild-type mice, it evoked PPI deficits in PACAP^+/−^ mice (Fig. 2A). Three-way ANOVA revealed a significant main effect of genotype [F_(1, 33)_ = 7.38, *p*<0.05] and treatment [F_(1, 33)_ = 10.0, *p*<0.01], a significant interaction between treatment and genotype [F_(1, 33)_ = 4.35, *p*<0.05], and no significant dB×genotype×treatment interaction [F_(2, 66)_ = 0.719, *p* = 0.49]. Multiple comparisons revealed that DOI (1 mg/kg) did not affect PPI in PACAP^+/+^ mice, whereas it significantly disrupted PPI compared with saline in PACAP^+/−^ mice (Fig. 2A). There were no significant differences in startle amplitudes between the four groups (PACAP^+/+^ and PACAP^+/−^ injected with saline or DOI) (Fig. 2B).

As it has been reported that stimulation of 5-HT_2A_ receptors activates hypothalamic neurons to increase the secretion of several hormones, such as corticosterone, and induces hypothermia [23], [24], we examined the effect of DOI on plasma corticosterone and body temperature in both genotypes. Although DOI dose-dependently increased the level of plasma corticosterone, there was no difference between the two genotypes (Fig. S3A). Repeated two-way ANOVA revealed a significant main effect of treatment [F_(3, 32)_ = 21.6, *p*<0.0001 ], but not genotype [F_(1, 32)_ = 0.011, *p* = 0.92]. Similarly, there was no difference in DOI-induced hypothermia between the two genotypes (Fig. S3B). Repeated two-way ANOVA revealed a significant main effect of treatment [F_(3, 39)_ = 16.6, *p*<0.001 ], but not genotype [F_(1, 39)_ = 0.193, *p* = 0.66].

### Identification of Brain Regions with an Altered Response to DOI in PACAP^+/−^ Mice

**Figure 3:**
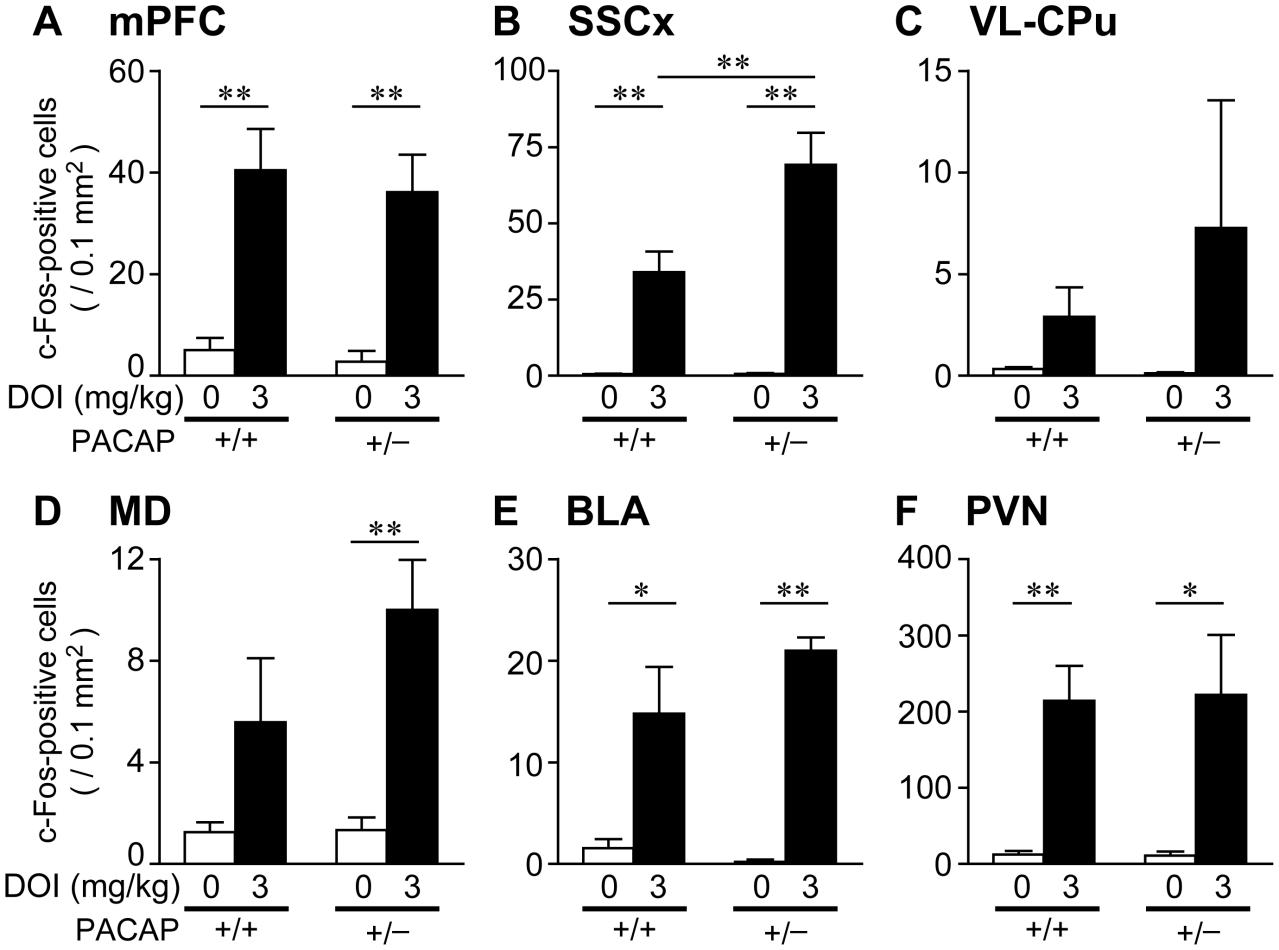
Effect of DOI on c-Fos expression. The number of c-Fos-positive cells in the mPFC (A), SSCx (B), VL-CPu (C), MD (D), BLA (E) and PVN (F) were determined in PACAP^+/+^ (+/+) and PACAP^+/−^ (+/−) mice after injection of DOI (3 mg/kg) or saline. Values are expressed as the mean ± SEM (n = 5–6). Statistically significant differences were assessed with two-way ANOVA with post hoc Tukey-Kramer test. *p<0.05, **p<0.01.

To identify the brain regions that may underlie the differences in behavioral response to DOI, we first examined DOI-induced changes in c-Fos protein expression in 16 brain regions known to be involved in the regulation of the head-twitch response, in PPI, in the release of corticosterone and the regulation of body temperature [25]–[28] in PACAP^+/+^ mice on the C57BL/6J×129S6/SvEvTac hybrid background (Fig. S1). Among the brain regions examined, significant differences in c-Fos expression were observed only in six; the mPFC, SSCx, VL-CPu, MD, BLA and PVN (Table 1). Therefore, we subsequently examined DOI-induced c-Fos expression in these six regions in PACAP^+/−^ mice. Representative photomicrographs of c-Fos staining in these regions are shown in Fig. S4. Interestingly, the number of c-Fos-positive cells was significantly increased specifically in the SSCx in PACAP^+/−^ mice compared with PACAP^+/+^ mice in response to DOI (Fig. 3 and S4). Two-way ANOVA revealed a significant main effect of treatment [F_(1, 18)_ = 54.2, *p*<0.001] and genotype [F_(1, 18)_ = 6.47, *p*<0.05], and a significant interaction between genotype and treatment for the SSCx [F_(1, 18)_ = 6.46, *p*<0.05]. In the remaining five regions (mPFC, VL-CPu, MD, BLA and PVN), there was no statistically significant difference in the number of c-Fos-positive cells after DOI injection between the two genotypes (Fig. 3).

**Table 1:** Effects of DOI on c-Fos expression in various brain regions in PACAP^+/+^ mice on the C57BL/6J×129S6/SvEvTac hybrid background.

The number of c-Fos-positive cells (per 0.1 mm^2^)			
Region	Saline	DOI (10 mg/kg)	*p* value
mPFC	14.4±4.0	41.6±6.5	0.008
Acb core	12.4±3.4	27.5±8.3	0.15
Acb shell	9.9±2.4	20.6±4.6	0.10
SSCx	4.7±2.8	58.6±14.2	0.012
DL-CPu	1.4±0.6	3.1±1.6	0.39
DM-CPu	14.4±4.9	25.3±6.1	0.21
VL-CPu	1.0±0.4	18.0±6.5	0.047
VP	7.1±2.2	12.2±3.0	0.30
BLA	9.0±2.9	25.6±3.9	0.015
LGP	0.8±0.4	1.9±0.6	0.26
MD	10.6±3.1	23.2±2.8	0.020
PVN	28.5±18.4	212.2±17.0	0.001
CA1	7.3±3.6	10.1±5.3	0.68
GrDG	7.5±3.2	7.5±3.1	0.99
PoDG	10.8±4.5	29.5±21.4	0.43
SNR	0.5±0.3	1.2±0.4	0.17

The regions are indicated in Figure S1. Data shows mean ± SEM of 4–6 mice. Statistically significant differences vs. saline were assessed with the *t*-test.

### Increase in the Number of c-Fos-positive Cells Among 5-HT_2A_ Receptor-negative Cells in the SSCx of PACAP^+/−^ Mice Injected with DOI

We next examined whether the difference between PACAP^+/+^ and PACAP^+/−^ mice in the molecular response of SSCx neurons to DOI was dependent on the expression levels of the 5-HT_2A_ receptor. 5-HT_2A_ receptor protein levels in the SSCx were similar in both genotypes as measured by Western blot analysis (Fig. S5).

**Figure 4:**
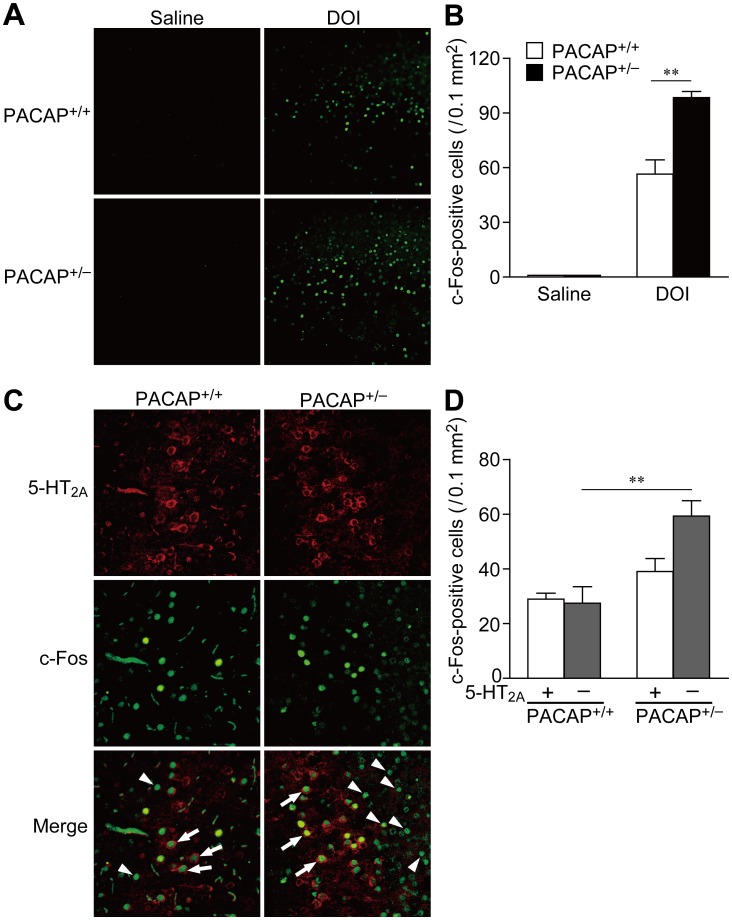
Number of c-Fos-positive/5-HT_2A_ receptor-negative cells is increased in the SSCx in PACAP^+/−^ mice. (A and B) Representative c-Fos immunofluorescence images (A) and quantitative data (B) in PACAP^+/+^ and PACAP^+/−^ mice. (C and D) Representative double-immunofluorescence images showing the co-localization of DOI-induced c-Fos and 5-HT_2A_ receptor immunoreactivity (C) and quantitative data (D) in PACAP^+/+^ and PACAP^+/−^ mice. Arrows indicate representative cells double-labeled for c-Fos and 5-HT_2A_ receptor, and arrowheads indicate those positive for c-Fos and negative for 5-HT_2A_ receptor. Values are expressed as the mean ± SEM (n = 4). Statistically significant differences were assessed with two-way ANOVA with post hoc Tukey-Kramer test. **p<0.01.

The types of neurons activated by DOI in the SSCx were then examined using double immunostaining for c-Fos and the 5-HT_2A_ receptor. The number of c-Fos-positive cells among 5-HT_2A_ receptor-positive cells was not changed in either genotype. However, the number of c-Fos-positive/5-HT_2A_ receptor-negative cells was significantly increased in the SSCx in PACAP^+/−^ mice compared with PACAP^+/+^ mice (Fig. 4). Two-way ANOVA revealed a significant main effect of genotype [F_(1, 12)_ = 18.6, P<0.001], but not 5-HT_2A_ co-expression [F_(1, 12)_ = 3.73, *p* = 0.078]. There was a significant interaction between genotype and 5-HT_2A_ co-expression [F_(1, 12)_ = 4.98, *p*<0.05].

## Discussion

In this study, we obtained four major findings. First, PACAP^+/−^ mice injected with DOI exhibit exaggerated head-twitch responses and PPI deficits, while PACAP^+/+^ mice with the same treatment show significantly reduced head-twitch responses and normal levels of PPI. Second, other 5-HT-induced responses mediated by 5-HT_2A_ receptor stimulation, such as increased corticosterone levels and hypothermia, did not significantly differ between PACAP^+/−^ and PACAP^+/+^ mice. Third, the DOI-induced increase in the number of c-Fos-positive cells was more pronounced in 5-HT_2A_ receptor-negative cells in the SSCx in PACAP^+/−^ mice compared with PACAP^+/+^ mice. Fourth, F_1_ PACAP^+/−^ mice from PACAP^+/−^ dams, but not from PACAP^+/+^ dams, show significant increases in locomotor activity, rearing time and time spent in the center of the open field compared with PACAP^+/+^ littermates.

We previously reported that PACAP^−/−^ mice exhibit an increased head-twitch response to an intermediate dose of DOI, 0.25 mg/kg body weight [4]. In addition, they display PPI deficits without receiving DOI injection [8]. In PACAP^+/−^ mice, we have not yet observed any appreciable behavioral changes. However, impaired long-term potentiation has been observed in the dentate gyrus in PACAP^+/−^ mice and in mice with altered expression of the PAC_1_ receptor [7], which have approximately 25% of the [^125^I]-PACAP27 binding density (due to a targeted deletion of exon 2) in their brain [29]. In addition, Ohtaki et al. [30] demonstrated that PACAP^+/−^ mice have an increased vulnerability to ischemic neuronal cell death, which is associated with decreased signal transducer and activator of transcription (STAT) 3 and extracellular signal-regulated kinase (ERK) activities. These observations imply that, given that the PACAP signaling system is a risk factor for psychiatric disorders, PACAP^+/−^ mice could serve as a useful model to assess vulnerability to non-genetic risk factors in subjects with genetic susceptibility.

Our present findings that PACAP^+/−^ mice display exaggerated head-twitch responses and PPI deficits, but not alterations in other parameters (such as corticosterone release or hypothermia) after DOI injection, suggest that these mice have a selective vulnerability to the hallucinogenic drug that specifically affects cortical sensory function. Indeed, among the brain regions examined (the mPFC, SSCx, VL-CPu, MD, BLA and PVN), the DOI-induced increase in the number of c-Fos-positive/5-HT_2A_ receptor-negative cells was more pronounced in the SSCx in PACAP^+/−^ mice compared with their wild-type littermates. Scruggs et al. [31] demonstrated that DOI activates 5-HT_2A_ receptors on thalamocortical neurons and thereby increases glutamate release, which in turn drives c-Fos expression in cortical glutamatergic neurons through a mechanism dependent on the ionotropic glutamatergic AMPA receptor. It may be possible that endogenious PACAP influences on the 5-HT_2A_–glutamate interactions. In our preliminary study, we have observed that PACAP modulates cell surface expression of 5-HT_2A_ receptors in heterologous cells. This effect of PACAP may explain susceptibility to hallucinogenic drugs in PACAP^+/−^ mice, although further studies (e.g. in neuronal cultures or in vivo brain via viral delivery) are necessary.

Hallucinogenic drugs have been shown to concomitantly induce both the head-twitch response and the expression of specific genes, including *egr-1* and *egr-2*, in the SSCx in a 5-HT_2A_ receptor-dependent manner [32]. The suppression of this system by activation of the metabotropic glutamate receptor 2 has been shown to abolish the hallucinogen-specific signaling and behavioral responses [33]. In support of this, we recently observed that a metabotropic glutamate 2/3 receptor agonist can reverse psychomotor abnormalities and recognition memory deficits in PACAP^−/−^ mice [34]. In a mouse model of maternal influenza viral infection, which is a risk factor for schizophrenia, an adult-onset abnormal response to DOI is observed, with an exaggerated head-twitch response and expression of the genes *c-fos*, *egr-1* and *egr-2* in cortical neurons [35]. It might be worth examining whether PACAP signaling pathways are altered in the mouse viral infection model [36].

The present observation that F_1_ PACAP^+/−^ mice from PACAP^+/−^ dams show behavioral abnormalities in the open field test may reflect genetic and environmental vulnerability in PACAP^+/−^ offspring. However, the potential genotypic impact of PACAP^+/−^ dams on the offspring might not be related to the changes in the head-twitch response, PPI deficits or SSCx activation observed in F_1_ PACAP^+/−^ mice from PACAP^+/+^ dams. Nonetheless, it will be interesting to investigate the neural and molecular mechanisms underlying the abnormalities in F_1_ PACAP^+/−^ mice obtained from PACAP^+/−^ dams. A future experiment, for example, using cross-fostering, should provide insight into the interactions between genetic and environmental risk factors.

In conclusion, we demonstrate that PACAP^+/−^ mice show specific vulnerability to the hallucinogenic drug DOI, which impacts cortical sensory function and results in exaggerated head-twitch responses and sensorimotor gating deficits. These findings suggest that the PACAP signaling pathway is critically involved in 5-HT_2_ receptor-dependent cortical processing. PACAP^+/−^ mice provide a promising model to investigate the neural and molecular mechanisms underlying impaired behavioral responses in which 5-HT_2_ receptors are involved.

## Supporting Information

Figure S1 **Diagrammatic representation of the brain regions examined for c-Fos expression.** The areas examined for counting c-Fos-positive cells include mPFC, Acb core, Acb shell, SSCx, DL-CPu, DM-CPu, VL-CPu, VP, BLA, LGP, MD, PVN, CA1, GrDG, PoDG and SNR (reproduced from Paxinos and Franklin’s the Mouse Brain in Stereotaxic Coordinates, 3rd Edition with permission of Elsevier).
(TIF)

Figure S2 **Effect of maternal genotype on PACAP^+/−^ mouse behavior in the open-field test.** Distance travelled (A, B), the number of rearings (C, D) and time spent in the center of the field (E, F) were determined as described in Methods S1 and are shown for PACAP^+/+^ (open circles) and PACAP^+/−^ (closed circles) mice. The mice were obtained from the intercross of male 129S6/SvEvTac PACAP^+/−^ and female C57BL/6J PACAP^+/+^ (A, C, E; n = 12–21) or PACAP^+/−^ (B, D, F; n = 20–32) mice. Values are expressed as the mean ± SEM. Statistically significant differences were assessed with two-way ANOVA followed by Tukey-Kramer test. *p<0.05, **p<0.01.
(TIF)

Figure S3 **Effect of DOI on plasma corticosterone levels and body temperature in PACAP^+/+^ and PACAP^+/−^ mice.** (A) Plasma corticosterone levels were determined (see Methods S1) in PACAP^+/+^ (open circles) and PACAP^+/−^ (closed circles) mice treated with the indicated doses of DOI or saline 30 min before the experiment. Values are expressed as the mean ± SEM (n = 4–7). (B) Body temperature was determined (see Methods S1) in PACAP^+/+^ (open columns) and PACAP^+/−^ (closed columns) mice treated with the indicated doses of DOI or saline. Changes in body temperature are indicated as the area under the curve. Corticosterone levels and body temperature were determined as described in Methods S1. Values are expressed as the mean ± SEM (n = 4–9). Statistically significant differences were assessed with two-way ANOVA with post hoc Tukey-Kramer test. *p<0.05, **p<0.01 vs. saline.
(TIF)

Figure S4 **Representative c-Fos immunohistochemistry images for PACAP^+/+^ and PACAP^+/−^ mice.** Representative images of c-Fos immunohistochemistry in the mPFC (A), SSCx (B), VL-CPu (C), MD (D), BLA (E), and PVN (F). Scale bars, 100 µm.
(TIF)

Figure S5 **5-HT_2A_ receptor protein levels in the SSCx are similar in both genotypes.** Expression levels of the 5-HT_2A_ receptor in the SSCx were determined using Western blot analysis as described in Methods S1.
(TIF)

Methods S1 (DOC)
