# Supplementary figures and images for: Increased Behavioral and Neuronal Responses to a Hallucinogenic Drug in PACAP Heterozygous Mutant Mice
Source: PLoS One. 2014 Feb 20;9(2):e89153. doi: 10.1371/journal.pone.0089153 (PMC3930680; doi:10.1371/journal.pone.0089153)

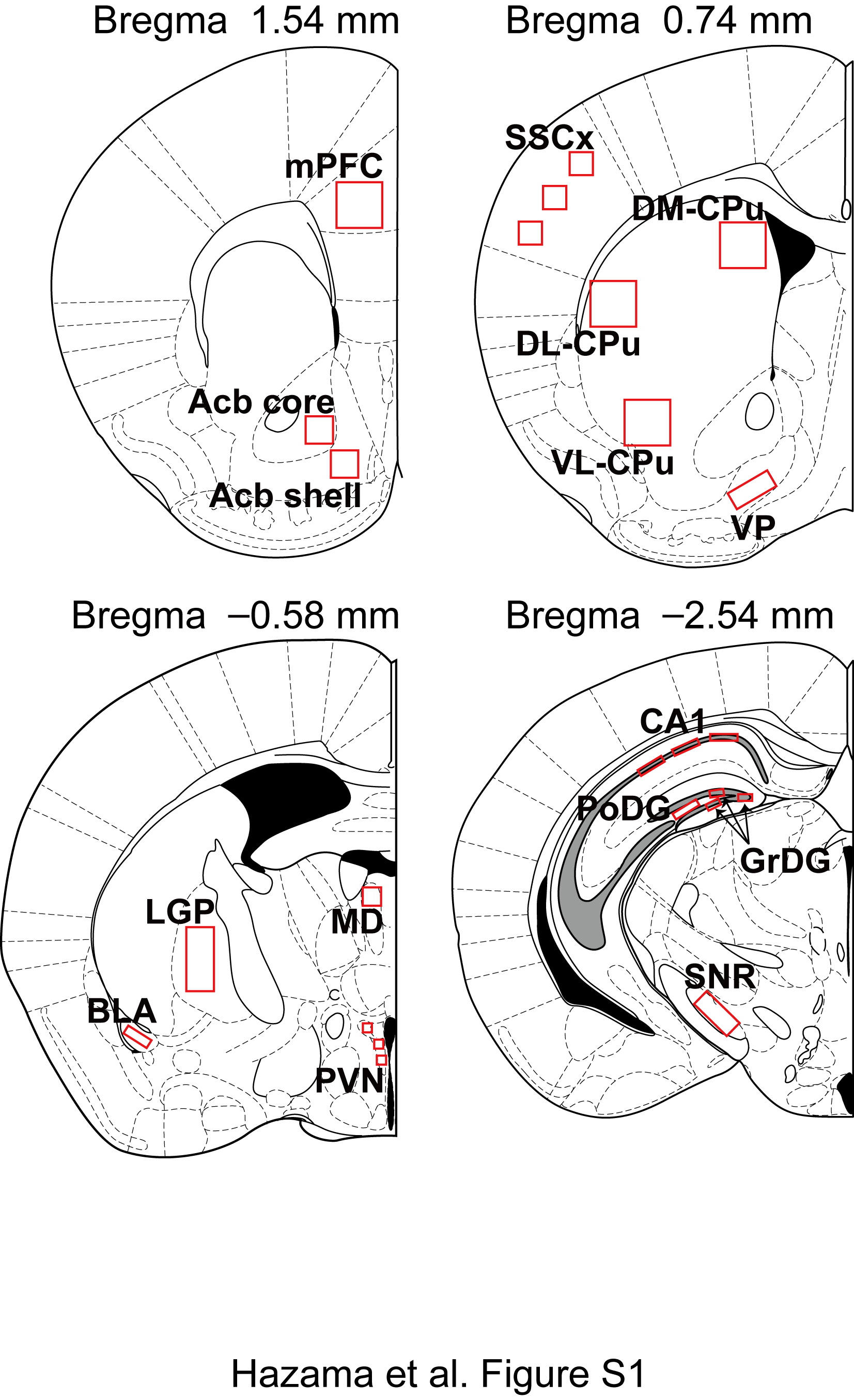

Supplement: Figure S1 — Diagrammatic representation of the brain regions examined for c-Fos expression. The areas examined for counting c-Fos-positive cells include mPFC, Acb core, Acb shell, SSCx, DL-CPu, DM-CPu, VL-CPu, VP, BLA, LGP, MD, PVN, CA1, GrDG, PoDG and SNR (reproduced from Paxinos and Franklin’s the Mouse Brain in Stereotaxic Coordinates, 3rd Edition with permission of Elsevier). (TIF) [file pone.0089153.s001.tif]

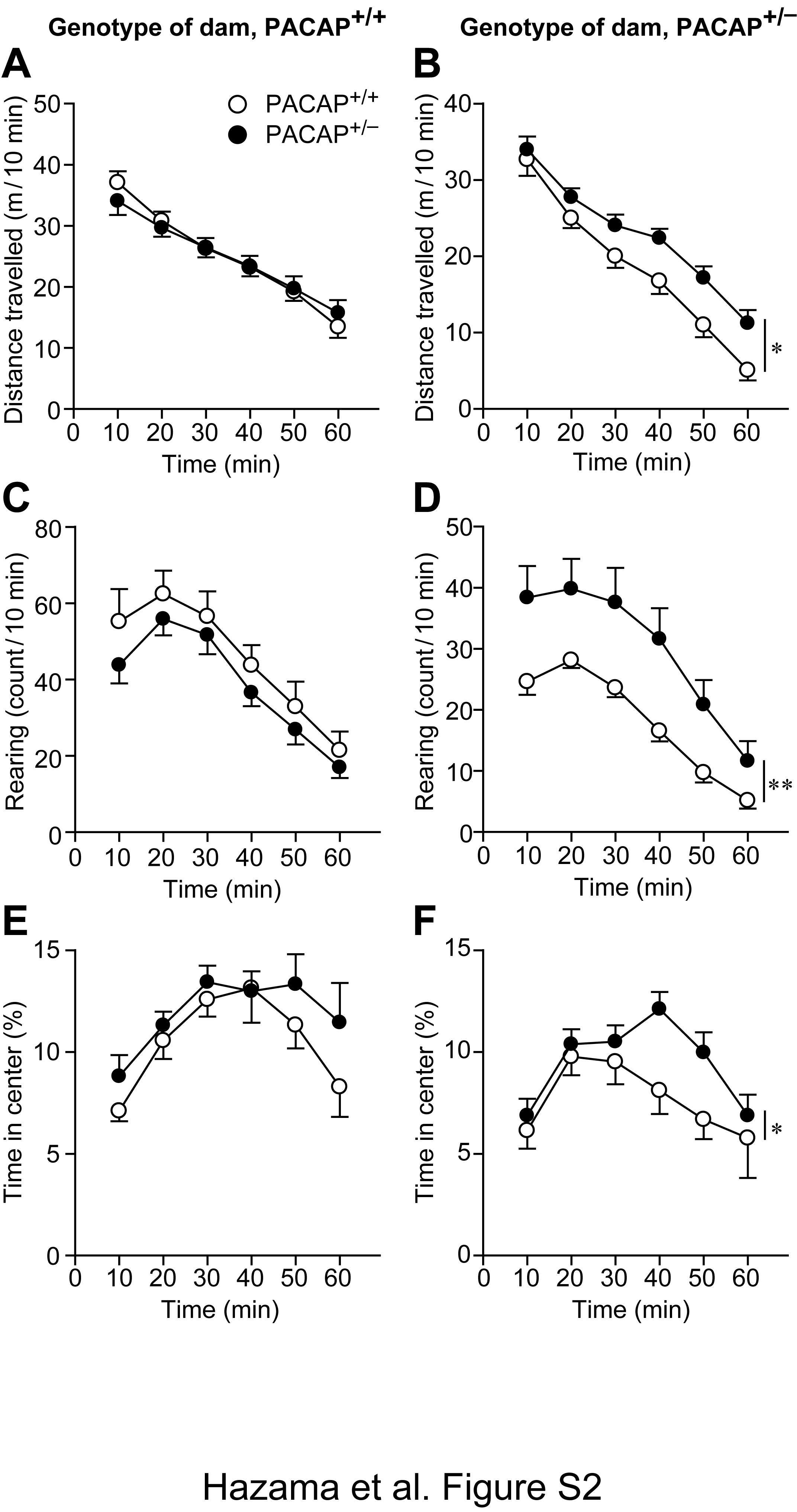

Supplement: Figure S2 — Effect of maternal genotype on PACAP+/− mouse behavior in the open-field test. Distance travelled (A, B), the number of rearings (C, D) and time spent in the center of the field (E, F) were determined as described in Methods S1 and are shown for PACAP+/+ (open circles) and PACAP+/− (closed circles) mice. The mice were obtained from the intercross of male 129S6/SvEvTac PACAP+/− and female C57BL/6J PACAP+/+ (A, C, E; n = 12–21) or PACAP+/− (B, D, F; n = 20–32) mice. Values are expressed as the mean ± SEM. Statistically significant differences were assessed with two-way ANOVA followed by Tukey-Kramer test. *p<0.05, **p<0.01. (TIF) [file pone.0089153.s002.tif]

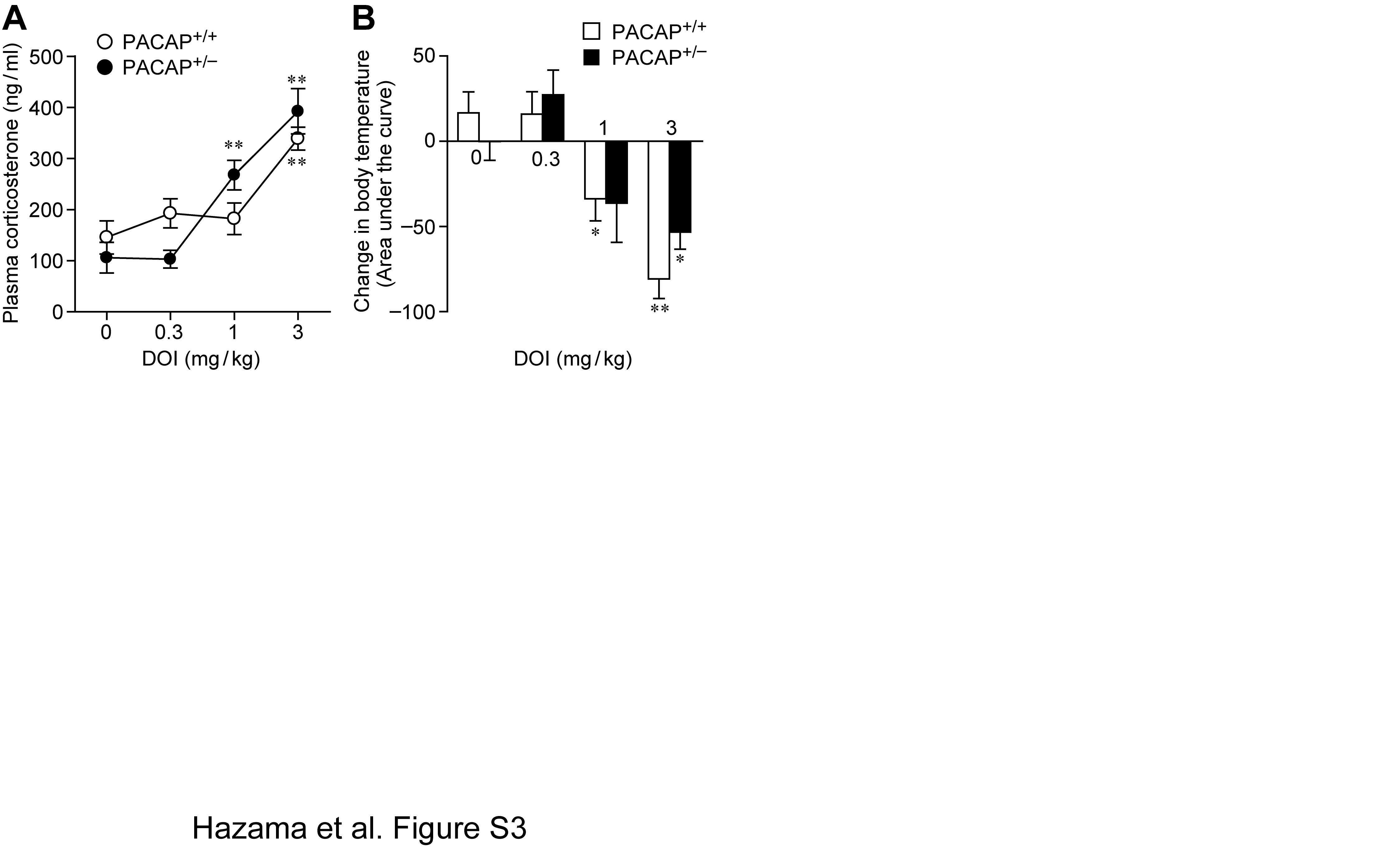

Supplement: Figure S3 — Effect of DOI on plasma corticosterone levels and body temperature in PACAP+/+ and PACAP+/− mice. (A) Plasma corticosterone levels were determined (see Methods S1) in PACAP+/+ (open circles) and PACAP+/− (closed circles) mice treated with the indicated doses of DOI or saline 30 min before the experiment. Values are expressed as the mean ± SEM (n = 4–7). (B) Body temperature was determined (see Methods S1) in PACAP+/+ (open columns) and PACAP+/− (closed columns) mice treated with the indicated doses of DOI or saline. Changes in body temperature are indicated as the area under the curve. Corticosterone levels and body temperature were determined as described in Methods S1. Values are expressed as the mean ± SEM (n = 4–9). Statistically significant differences were assessed with two-way ANOVA with post hoc Tukey-Kramer test. *p<0.05, **p<0.01 vs. saline. (TIF) [file pone.0089153.s003.tif]

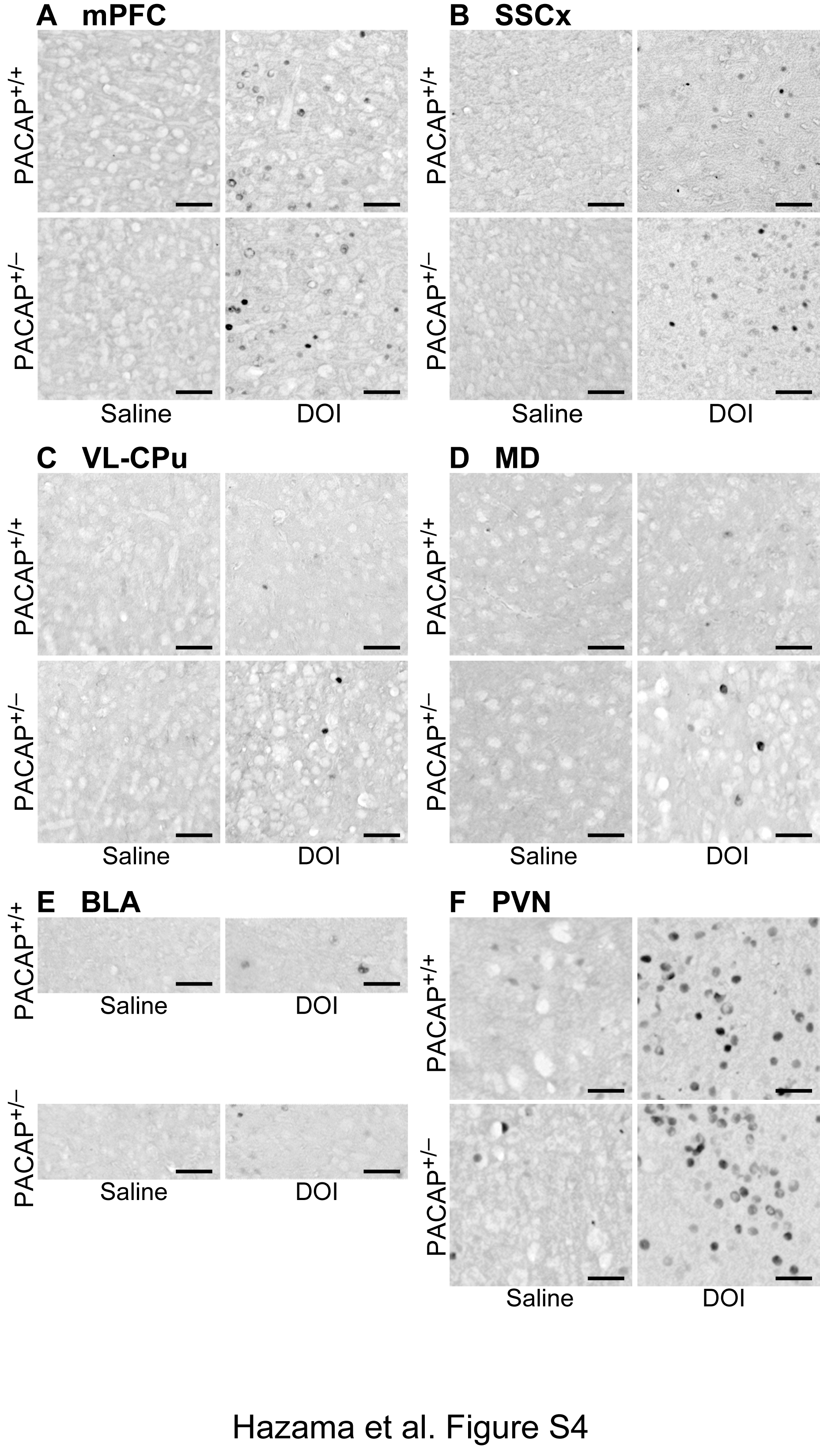

Supplement: Figure S4 — Representative c-Fos immunohistochemistry images for PACAP+/+ and PACAP+/− mice. Representative images of c-Fos immunohistochemistry in the mPFC (A), SSCx (B), VL-CPu (C), MD (D), BLA (E), and PVN (F). Scale bars, 100 µm. (TIF) [file pone.0089153.s004.tif]

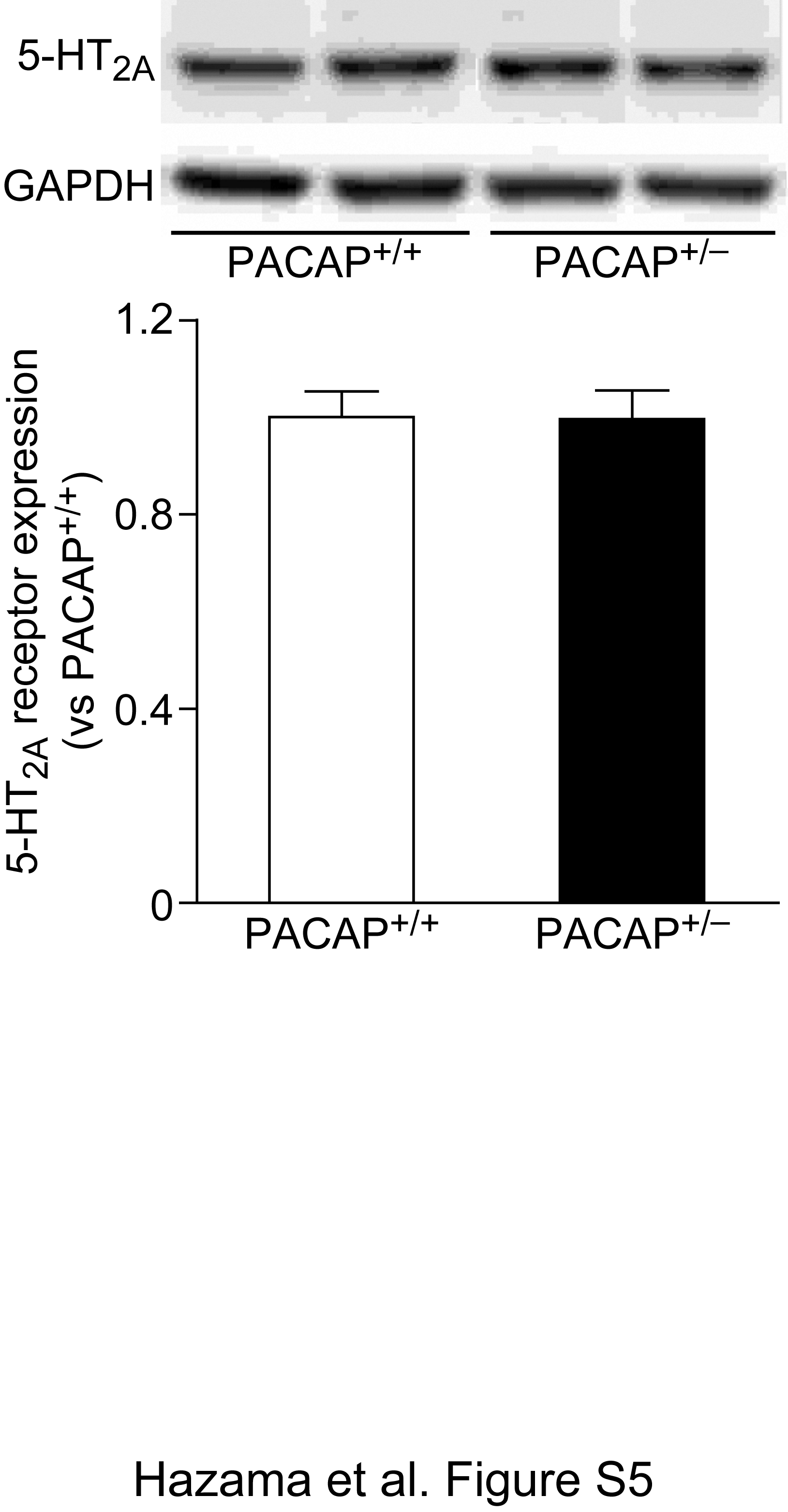

Supplement: Figure S5 — 5-HT2A receptor protein levels in the SSCx are similar in both genotypes. Expression levels of the 5-HT2A receptor in the SSCx were determined using Western blot analysis as described in Methods S1. (TIF) [file pone.0089153.s005.tif]
