# Supplementary material for: Increased Behavioral and Neuronal Responses to a Hallucinogenic Drug in PACAP Heterozygous Mutant Mice
Source: PLoS One. 2014 Feb 20;9(2):e89153. doi: 10.1371/journal.pone.0089153 (PMC3930680; doi:10.1371/journal.pone.0089153)
Supplement: Methods S1 — (DOC) [file pone.0089153.s006.doc]

**Increased Behavioral and Neuronal Responses to a Hallucinogenic Drug in PACAP Heterozygous Mutant Mice**

**Supplementary Methods**

Locomotor activity

Locomotor activity in an open field was quantified using an infrared photocell beam detection system, Acti-Track (Panlab, Barcelona, Spain). In this study, a clear plastic box (45 × 45 × 15 cm) was used for locomotor measurement. Each mouse was placed individually in a clear plastic box (45 × 45 × 15 cm) and tracked for 60 min. The center of the field was defined as a 22.5 × 22.5 cm square.

Corticosterone Measurements

Mice were administrated intraperitoneally with either saline or DOI (0.3−3 mg/kg). Thirty minutes after injection, trunk blood was collected into 1.5-ml plastic tubes containing 5 µl of 0.5 M ethylenediaminetetraacetic acid (EDTA). Samples were immediately centrifuged and heated at 60 °C for 30 min, and the plasma was stored at −80 °C until assayed. Corticosterone levels were determined with the Rat Corticosterone ^125^I Biotrack Assay System (GE Healthcare, Piscataway, NJ).

Measurement of Rectal Body Temperature

Rectal body temperature of mice was measured using a BAT-12 digital thermometer coupled with a RET-3 rectal probe (Physitemp Instruments Inc., Clifton, NJ).

Western Blot Analysis

The SSCx was harvested and homogenized in cold extraction buffer (20 mM Tris-HCl, pH 7.4, 1 mM EDTA, 0.15 M NaCl, 1% Nonidet P-40, 5% glycerol, 5 mM L-mercaptoethanol, 1 mM phenylmethylsulfonyl fluoride, 1 μg/ml aprotinin, 1 μg/ml leupeptin and 1 mM dithiothreitol). The homogenized samples were centrifuged at 6,200 × g for 15 min at 4 °C, and the supernatants were recovered, the protein concentrations of which were determined with the BCA protein assay kit (Pierce Biotechnology Inc., Rockford, IL). Twenty micrograms of the sample protein was resolved by 10% SDS-PAGE and transferred to a polyvinylidene difluoride membrane (Millipore, Tokyo, Japan). The membrane was incubated in 50 mM Tris-HCl (pH 7.4) and 0.05% Tween-20 (TBST) containing 5% bovine serum albumin for 1 h at room temperature to block non-specific binding. The membrane was then incubated for another 24 h at 4 °C with rabbit anti-5-HT_2A_ receptor polyclonal antibody (1:1 000; Abcam) or with mouse anti-GAPDH polyclonal antibody (1:10 000; Millipore). After the membrane was washed in TBST, it was incubated with species-specific secondary antibodies (goat anti-rabbit peroxidase-conjugated antibody [1:1 000; Cappel, Durham, NC] and goat anti-mouse peroxidase-conjugated antibody [1:2 000; Cappel]) for 1 h at 22 °C. After washing in TBST, immunoreactive bands were visualized by incubation with an enhanced chemiluminescence substrate (Western Lightning Chemiluminescence Reagent Plus; PerkinElmer, Boston, MA). Detection and quantitation of relative band densities was performed using an ImageQuant LAS 4010 (GE Healthcare).
